# *In vivo* Evidence for Brain Region-Specific Molecular Interactions Between Cannabinoid and Orexin Receptors

**DOI:** 10.3389/fnins.2021.790546

**Published:** 2021-12-21

**Authors:** Hye Ji J. Kim, Ayat Zagzoog, Anna Maria Smolyakova, Udoka C. Ezeaka, Michael J. Benko, Teagan Holt, Robert B. Laprairie

**Affiliations:** ^1^College of Pharmacy and Nutrition, University of Saskatchewan, Saskatoon, SK, Canada; ^2^Department of Pharmacology, College of Medicine, Dalhousie University, Halifax, NS, Canada

**Keywords:** cannabinoid, cannabinoid receptor, receptor antagonist, orexin receptor, heterodimerization, colocalization, tetrad analysis, ventral striatum

## Abstract

The endocannabinoid and orexin neuromodulatory systems serve key roles in many of the same biological functions such as sleep, appetite, pain processing, and emotional behaviors related to reward. The type 1 cannabinoid receptor (CB1R) and both subtypes of the orexin receptor, orexin receptor type 1 (OX1R) and orexin receptor type 2 (OX2R) are not only expressed in the same brain regions modulating these functions, but physically interact as heterodimers in recombinant and neuronal cell cultures. In the current study, male and female C57BL/6 mice were co-treated with the cannabinoid receptor agonist CP55,940 and either the OX2R antagonist TCS-OX2-29 or the dual orexin receptor antagonist (DORA) TCS-1102. Mice were then evaluated for catalepsy, body temperature, thermal anti-nociception, and locomotion, after which their brains were collected for receptor colocalization analysis. Combined treatment with the DORA TCS-1102 and CP55,940 potentiated catalepsy more than CP55,940 alone, but this effect was not observed for changes in body temperature, nociception, locomotion, or *via* selective OX2R antagonism. Co-treatment with CP55,940 and TCS-1102 also led to increased CB1R-OX1R colocalization in the ventral striatum. This was not seen following co-treatment with TCS-OX2-29, nor in CB1R-OX2R colocalization. The magnitude of effects following co-treatment with CP55,940 and either the DORA or OX2R-selective antagonist was greater in males than females. These data show that CB1R-OX1R colocalization in the ventral striatum underlies cataleptic additivity between CP55,940 and the DORA TCS-1102. Moreover, cannabinoid-orexin receptor interactions are sex-specific with regards to brain region and functionality. Physical or molecular interactions between these two systems may provide valuable insight into drug-drug interactions between cannabinoid and orexin drugs for the treatment of insomnia, pain, and other disorders.

## Introduction

Endocannabinoid and orexin interdependence has been a topic of growing interest in the last two decades. Within the endocannabinoid system (ECS), lipid-based agonists, anandamide (AEA) and 2-arachidonoylglycerol (2-AG) primarily bind to 2 subtypes of the cannabinoid G protein-coupled receptors (GPCR), cannabinoid receptor type 1 (CB1R) and cannabinoid receptor type 2 (CB2R). The orexin system shares many physical and functional similarities with the ECS (Berrendero et al., 2018). It consists of the neuropeptides orexin A (OXA) and orexin B (OXB), which activate GPCRs, orexin receptor type 1 (OX1R) and type 2 (OX2R). Cannabinoid and orexin receptors are found in many of the same brain regions underlying complex behaviors such as sleep, appetite, and reward processing. In mice and humans, cannabinoid receptor (CBR) activation leads to sedative effects such as catalepsy, hypothermia, analgesia, and anti-locomotion (Metna-Laurent et al., 2017; Zagzoog et al., 2020, 2021). Orexin receptor activation increases arousal, body temperature, and modulates anti-nociception at the spinal and supraspinal levels (Yamanaka et al., 2003; Monda et al., 2005; Chiou et al., 2010). Dual orexin receptor antagonists (DORA) are emerging as safe and effective treatments for insomnia (Herring et al., 2019), while phytocannabinoids such as cannabidiol and Δ^9^-tetrahydrocannabinol (Δ^9^-THC), are used as off-label sleep aids as they affect the same sleep-wake neuropathways (Babson et al., 2017). Molecular and cellular interactions between these neuromodulatory systems have physiological implications in homeostasis, neurological and psychiatric disorders, as well as in drug-drug interactions between cannabinoid and orexin drugs.

Evidence for physical interactions between the endocannabinoid and orexin systems lies in the colocalization and potential heterodimerization of these two system’s receptors (Jäntti et al., 2014; Berrendero et al., 2018). CB1R co-localizes with both OX1R and OX2R in the neocortex, hippocampus, thalamus, hypothalamus, amygdala, ventral tegmental area, periaqueductal gray, dorsal raphe nucleus, and deep cerebellar nuclei (Marcus et al., 2001; Mackie, 2005; Flores et al., 2014). Bioluminescence resonance energy transfer studies have found that OX1R and OX2R are capable of forming homo- and heterodimeric complexes with one another and with CB1R (Jäntti et al., 2014). Moreover, fluorescence resonance energy transfer imaging demonstrates that CB1R-OX1R heterodimers reside in intracellular vesicles following CB1R agonist-mediated receptor internalization (Ellis et al., 2006; Ward et al., 2011). In recombinant cells co-expressing these receptor subtypes, OX1R activity not only induces 2-AG synthesis, but it also potentiates extracellular-signal-regulated kinase (ERK) activity of these recombinant cells (Jäntti et al., 2014). The reverse has been shown, as CB1R activation increases OXA’s potency to activate ERK in cells where CB1R and OX1R are co-localized and potentially heterodimerized (Hilairet et al., 2003). In addition to recombinant cells, CB1R-OX1R complexes have also been observed in embryonic mouse hypothalamic neurons, supporting the workings of these heterodimers *in vivo* (Imperatore et al., 2016).

Although CB1R-OX1R heterodimers have not previously been directly observed in animals, rodent studies in which cannabinoid and orexin compounds are co-administered have described unique physiological and behavioral outcomes based on (1) the receptor subtype targeted, and (2) separate versus combined compound treatments. Activating CBRs while blocking orexin receptors causes sedation or sleep-like effects (Flores et al., 2016; Petrunich-Rutherford and Calik, 2021). In contrast, acutely activating both cannabinoid and orexin receptors increases appetite (Mechoulam and Fride, 2001; Merroun et al., 2015) and reward sensitivity (Plaza-Zabala et al., 2012; Flores et al., 2014; Yazdi et al., 2015). Thus, cannabinoid and orexin receptors may potentiate one another in brain regions modulating appetite and reward, while having antagonistic interactions in regions underlying arousal and sleep. Dual control of these biological functions is receptor-subtype specific. Physiological and behavioral regulation of body temperature, nociception processing, locomotion, appetite, and cognition are thought to be primarily CB1R-dependent based on the higher abundance of CB1R compared to CB2R (Zanettini et al., 2011). Between the orexin receptor subtypes, persistent OX2R activity is believed to be more critical for maintaining arousal (Willie et al., 2003; Mieda et al., 2011) and caloric homeostasis (Funato et al., 2009). The combined observations of heterodimerization *in vitro*, existence in the same brain regions *in vivo*, and overlapping physiological effects of the ECS and orexin system support the hypothesis that co-manipulation of both systems will produce a fundamentally different outcome than targeting either system alone.

## Materials and Methods

### Compounds

CP55,940 (Cat # 90084) and TCS-1102 (Cat # 18495) were purchased from Cayman Chemical Company (Ann Arbor, MI). TCS-OX2-29 was purchased from Abcam (Waltham, MA, Cat # 141316). All compounds were stored at −20°C until use. CP55,940 was first dissolved in 100% methanol, then added to a vehicle solution consisting of: 1 part ethanol, 1 part Kolliphor EL (MilliporeSigma, Oakville), and 18 parts 1 M phosphate-buffered saline (PBS) (Fisher, Waltham, MA). The concentration of stock solution used for CP55,940 varied based on animal weight and treatment dose. TCS-OX2-29 and TCS-1102 were first dissolved in a 10% DMSO solution in PBS, then added to a vehicle solution consisting of 1 part ethanol, 1 part Kolliphor EL, and 18 parts PBS. TCS-OX2-29 was prepared as a 5 mg/mL stock solution. TCS-1102 was prepared as a 1.5 mg/mL stock solution. All compounds were prepared at room temperature, after which they were stored at 4°C overnight before use the next morning.

### Animals and Tetrad Testing

Adult male and female C57BL/6 mice aged 6–12 weeks (mean weight of males: 22 ± 0.3 g; mean weight of females: 20 ± 0.3 g) were purchased from Charles River Labs (Senneville, QC). Mice were group housed (males: 3 per cage; females: 5 per cage) with *ad libitum* access to food, water, and environmental enrichment. All mice were maintained on a 12 h light:dark cycle (07:00-19:00/19:00-07:00). Mice were randomly designated to receive 2 intraperitoneal (i.p.) injections (1 on each side) of the following treatment combinations: vehicle and CP55,940 at 5 doses (0.1, 0.3, 1, and 3 mg/kg), vehicle and TCS-OX2-29 at 4 doses (1, 10, 18, and 30 mg/kg), vehicle and TCS-1102 at 4 doses (0.1, 0.3, 1, and 10 mg/kg), 1 mg/kg CP55,940 and TCS-OX2-29 (1, 10, 18, and 30 mg/kg) at 4 doses, or 1 mg/kg CP55,940 and TCS-1102 (0.1, 0.3, 1, and 10 mg/kg) at 4 doses, totaling 22 treatment groups (*n* = 6 per group). CP55,940 doses were based on previously published studies from our group in the same battery of *in vivo* assays (Zagzoog et al., 2020, 2021). TCS-OX2-29 doses were chosen to build on the work of Flores et al. (2016), who had tested 10 mg/kg of TCS-OX2-29 in mice. Because TCS-1102 has not previously been assessed in the tetrad, doses of TCS-1102 were also chosen based on Flores et al. (2016). For combination treatments, 1 mg/kg CP55,940 was chosen as an approximation of the ED_80_ for this compound. These doses were piloted by our group for safety and effect prior to data collection for the current study. These 22 treatment groups were tested in both males and females, totaling 264 mice used throughout the study. All protocols were in accordance with the guidelines detailed by the Canadian Council on Animal Care (CCAC; Ottawa ON: Vol. 1, second Ed., 1993; Vol. 2, 1984) and approved by the Animal Research Ethics Board and the Scientific Merit Review Committee for Animal Behavior at the University of Saskatchewan. In keeping with the Animal Research: Reporting of *In Vivo* Experiments (ARRIVE) guidelines, power analyses were conducted to determine the minimum number of mice required for the study, and mice were purchased, rather than bred, to limit animal waste (Kilkenny et al., 2010).

Tetrad testing commenced 10 min after i.p. injections with the ring holding assay to measure catalepsy. For this assay, mice were placed on the ring apparatus such that their forepaws clasped the 5 mm ring positioned 5 cm above the surface of the testing platform. The length of time that the ring was clasped was recorded (s). The trial was completed when the mouse turned its head or body, made 3 consecutive escape attempts, or at 60 s of immobility [i.e., maximum possible effect (MPE) = 60 s]. Internal body temperature was recorded 15 min after the injections *via* a rectal thermometer (°C). Thermal anti-nociception was assessed by the tail flick latency test 20 min following the injections. Mice were restrained with their tails placed ∼ 1 cm into 52 ± 2°C water. The time until the tail was removed from the water was recorded as the tail flick latency (s). Tails were removed after 20 s if they had not been removed already (i.e., MPE = 20 s). Locomotion was measured in the open field test 25 min following the injections. Mice were placed in the 55 cm × 55 cm square-shaped open field for 10 min, during which they were free to roam. Distance traveled (m) and average velocity (cm/s) were measured with EthoVision XT (Noldus Information Technology Inc., Leesburg, VA). Distance and velocity scores were then normalized and expressed as a percentage of vehicle means (%Vehicle). For statistical processing, tetrad scores were averaged between mice in the same drug treatment group (Zagzoog et al., 2020, 2021).

### Tissue Perfusion and Immunohistochemistry

A separate set of mice were euthanized, and their brains were collected 30 min after i.p. injections. This tissue collection time was based on the tetrad timeline, as treated mice finished the tetrad test 30–35 min post-injection. Mice were placed in a rodent vapor chamber, which delivered a mixture of oxygen and isoflurane for approximately 2 min before the animal was fully anesthetized. Mice were then transcardially perfused with 5 mL of ice cold 0.9% saline solution, followed by 5 mL of ice cold 4% paraformaldehyde solution. Brains were rapidly dissected from the skull then submerged in 4% paraformaldehyde solution on ice. The perfused mouse brains were then stored at 4°C for 1 day before being submerged in 30% sucrose solution and stored at 4°C for another 1–1.5 days. Once the brains sunk to the bottom of the sucrose solution, the sucrose solution was drained, and the brains underwent flash-freezing using liquid nitrogen. Frozen brains were stored at −80°C prior to slicing. For slicing, frozen brains were embedded in Tissue-Plus*™*. C.T. Compound (Fisher, Waltham, MA), then sliced at a thickness of 20 μm using a cryostat held at −20°C. Slices were mounted on Superfrost*™* Plus microscope slides (Fisher, Waltham, MA) then stored at −20°C until they were used for immunohistochemistry.

The immunohistochemistry procedure consisted of the following steps: (1) blocking endogenous peroxidase by incubating with 0.3% H_2_O_2_ at room temperature for 10 min, (2) rinsing in 1 M PBS 3 times for 5 min each time, (3) blocking non-specific binding at room temperature by incubating in 10% fetal bovine serum at room temperature for 2 h, (4) incubating with primary antibodies at 4°C for 24 h, (5) rinsing in 1 M PBS 3 times for 5 min each, (6) incubating in secondary antibodies at room temperature for 1 h, at which point all steps proceeded in the dark due to the secondary antibodies’ light sensitivity, (7) rinsing in 1 M PBS 3 times for 5 min each, and (8) mounting the immuno-stained slices using ProLong*™* Gold Antifade Mountant with DAPI (ThermoFisher Scientific, Waltham, Massachusetts). All immune-stained slices were stored at 4°C before imaging. The following primary antibodies were used: cannabinoid receptor CB1R monoclonal antibody (mouse, Synaptic Systems, Göttingen, Germany, Lot 1–3, Cat # 258011) diluted at 1:500, orexin receptor 1 polyclonal antibody (rabbit, Enzo Life Sciences, Farmingdale, NY, Lot 10122020, Cat # BML-KI508) diluted at 1:50, orexin receptor 2 polyclonal antibody (rabbit, Enzo Life Sciences, Farmingdale, NY, Lot 10122020, Cat # BML-KI507) diluted at 1:200. The following secondary antibodies were used: Goat Anti-Mouse IgG H&L (Alexa Fluor^®^ 594) (Invitrogen, Eugene, Oregon, Lot 2179228, Cat # A11005) diluted at 1:500 was used for the mouse anti-CB1R primary antibody, while Goat Anti-Rabbit IgG H&L (Alexa Fluor^®^ 488) (Invitrogen, Eugene, Oregon, Lot 2179202, Cat # A11008) diluted at 1:500 was used for the rabbit-anti-OX1R and -OX2R antibodies. Each brain region was triple-labeled with (1) DAPI-CB1R-OX1R or (2) DAPI-CB1R-OX2R.

### Confocal Microscopy and Colocalization Analysis

A Zeiss LSM700 confocal microscope (Carl Zeiss, Oberkochen, Germany) equipped with Zeiss ZEN Black (version 2.3 SP1) software (Carl Zeiss, Oberkochen, Germany) was used to obtain fluorescent 3D images of the immuno-stained brain slices. 10–12 Z-stacks encompassing an average tissue depth of 15 μm were collected at 63X oil immersion from each ventral striatum and primary motor cortex. ImageJ (version 2.1.0) (NIH, Bethesda, MD, United States) was used to merge the Z-stacks to form 3D images for analysis. Within each image, DAPI-immunolabeled cells were randomly chosen for colocalization analysis on ImageJ and its Fiji package (NIH, Bethesda, MD, United States). Pearson’s correlation coefficients were calculated for (1) CB1R-OX1R, and (2) CB1R-OX2R (Zinchuk and Zinchuk, 2008). For statistical processing, CB1R-OX1R and CB1R-OX2R colocalization coefficients were averaged between *n* = 6 cells in the same sex and drug treatment group.

### Statistical Analysis

Tetrad data are presented as mean ± SEM where “n” represents the number of animals per treatment group. Data from the ring holding assay and tail flick assay are reported as percent maximum possible effect (%MPE) for catalepsy and %MPE for anti-nociception, respectively. Results from the open field test are stated as a percentage of vehicle scores (%Vehicle). Dose-response curves were fit using a three parameter non-linear regression to yield the *ED*_50_ and *E*_*max*_ values (GraphPad, Prism, v. 9.0.1, San Diego, CA). For data without a clear dose-response (i.e., non-converged/“n.c.”), *E*_*max*_ was reported as the maximum response observed. All *E*_*max*_ data are reported as mean ± SEM. *ED*_50_ data are reported as the mean with 95% confidence interval (CI). Isobolographic analyses for body temperature were conducted using *ED*_50_ data with 95% CI only because *ED*_50_ could not be estimated in other data sets. Homogeneity of variance was confirmed using Bartlett’s test. Statistical analyses for tetrad data were conducted by two-way analysis of variance (ANOVA) to account for both sex and drug treatment. Immunohistochemistry colocalization data are presented as a mean ± SEM where “n” represents individual cells counted within the brain region of a single mouse. Colocalization means are denoted as Pearson’s correlation coefficients. Statistical analyses for the colocalization data were conducted by two-way ANOVA to account for both sex and drug treatment. *Post hoc* analyses were performed using Tukey’s (two-way ANOVA) test. Significance was set as *p* < 0.05.

## Results

### Catalepsy

**FIGURE 1:**
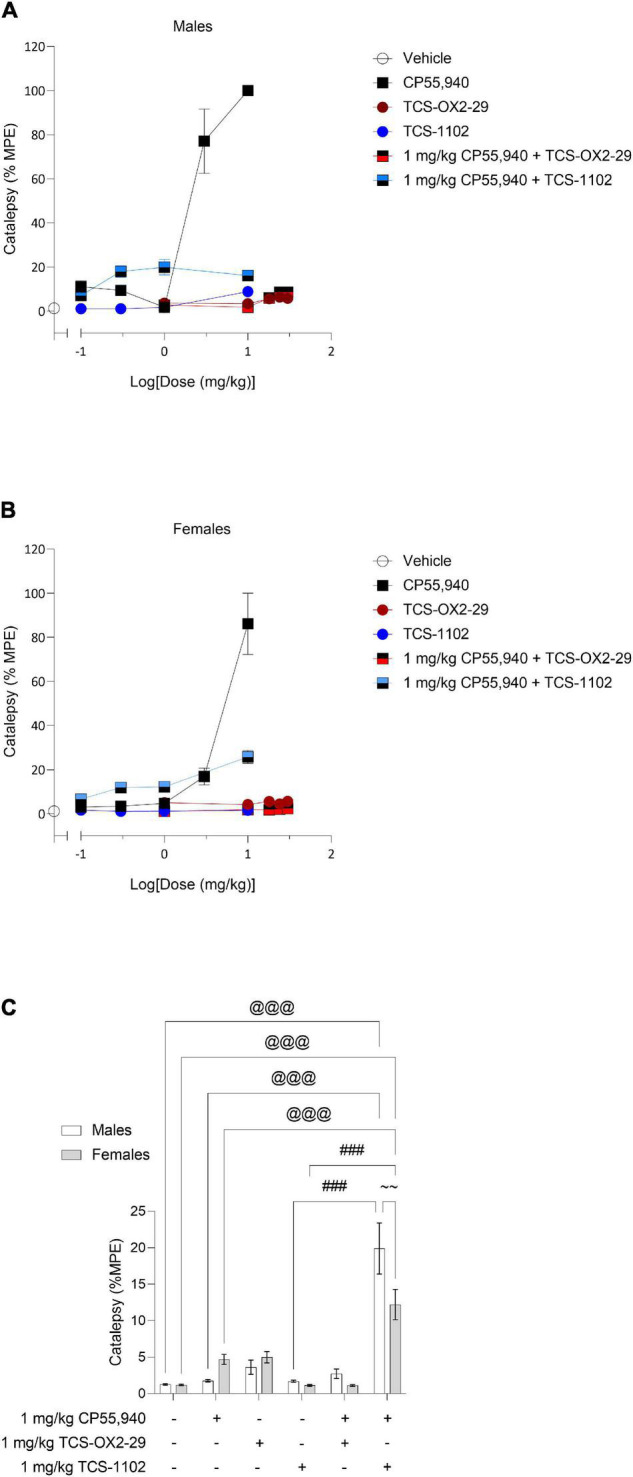
Acute catalepsy effects following cannabinoid and orexin drug treatments. Male **(A)** and female **(B)** C57BL/6 mice aged 6–12 weeks were i.p. administered one of the following dose ranges: CP55,940 (0.1–10 mg/kg), TCS-OX2-29 (1–30 mg/kg), TCS-1102 (0.1–10 mg/kg), 1 mg/kg CP55,940 + TCS-OX2-29 (1–30 mg/kg), or 1 mg/kg CP55,940 + TCS-1102 (0.1–10 mg/kg). 10 min post-injections, mice were assessed for catalepsy in the ring holding assay. **(C)** Cataleptic responses were compared within (sex) and between (drugs) the following experimental groups: 1 mg/kg CP55,940, 1 mg/kg TCS-OX2-29, 1 mg/kg TCS-1102, 1 mg/kg CP55,940 + 1 mg/kg TCS-OX2-29, or 1 mg/kg CP55,940 + 1 mg/kg TCS-1102. All catalepsy data are expressed as %MPE (MPE = 60 s), and as means ± SEM. *n* = 6 for all treatment groups. Significance was calculated using a two-way ANOVA followed by Tukey’s *post hoc* analyses. @@@ *p* < 0.001 compared to Vehicle within sexes. ###*p* < 0.001 compared between 1 mg/kg TCS-1102 and 1 mg/kg CP55,940 + 1 mg/kg TCS-1102. ∼∼*p* < 0.01 compared between sexes, within treatment groups.

Male and female C57BL/6 mice were treated with CP55,940 (0.1–10 mg/kg), TCS-OX2-29 (1–30 mg/kg), TCS-1102 (0.1–10 mg/kg), and co-treatments of 1 mg/kg CP55,940 and either TCS-OX2-29 (1–30 mg/kg) or TCS-1102 (0.1–10 mg/kg). Treatment with CP55,940 alone or 1 mg/kg CP55,940 + TCS-1102 produced a dose-dependent increase in catalepsy in both sexes (Figures 1A,B). Co-treatment with 1 mg/kg CP55,940 + TCS-1102 was less potent in producing catalepsy compared to CP55,940 alone in both sexes (Table 1). There were no sex differences within drug treatments. Potency differences could not be calculated between or within these other experimental groups because no clear dose-response was observed for all treatments (Table 1). Co-treatments of 1 mg/kg CP55,940 + TCS-OX2-29 or 1 mg/kg CP55,940 + TCS-1102 were less efficacious than 1 mg/kg CP55,940 alone (*p* < 0.001) (Table 1). Looking at the TCS-1102 treatments in females, co-treatment with 1 mg/kg CP55,940 + TCS-1102 was more efficacious than TCS-1102 alone (*p* = 0.0159) (Table 1). There were no efficacy differences between any of the other drug treatments, nor between sexes.

**TABLE 1:** *ED*_50_ and *E*_*max*_ values reflecting catalepsy responses to cannabinoid and orexin drug treatments.

Treatment	*ED*_50_ (mg/kg) (95% CI)	*E*_*max*_ (%MPE) ± SEM
	
	Males	
CP55,940	2.4 (1.3–4.3)	100
TCS-OX2-29	n.c.	5.8 ± 0.83
TCS-1102	n.c.	8.8 ± 1.2
1 mg/kg CP55,940 + TCS-OX2-29	n.c.	8.6 ± 0.52*
1 mg/kg CP55,940 + TCS-1102	41 (19–87)*	19 ± 1.5*

	**Females**	
CP55,940	5.7 (3.6–9.3)	86 ± 14
TCS-OX2-29	n.c.	5.7 ± 1.2
TCS-1102	n.c.	1.6 ± 0.29
1 mg/kg CP55,940 + TCS-OX2-29	n.c.	2.8 ± 1.5*
1 mg/kg CP55,940 + TCS-1102	26 (18–38)*	26 ± 3.0*#

*Data were fit to a three parameter non-linear regression with a system minimum and maximum constrained to 0 and 100, respectively (GraphPad, Prism, v. 8.0).*

*n.c., not converged.*

*For data without a clear dose–response (i.e., “n.c.”), E_max_ is reported as the maximum response observed.*

*Data are expressed as mg/kg with 95% CI or %MPE ± SEM.*

*p < 0.05 compared to CP55,940 within sexes, and #p < 0.05 compared between 1 mg/kg TCS-1102 and 1 mg/kg CP55,940 + 1 mg/kg TCS-1102, as determined by non-overlapping 95% CI or two-way ANOVA followed by Tukey’s post hoc test. Corresponding graph is presented in Figure 1.

Co-treatment with 1 mg/kg CP55,940 + 1 mg/kg TCS-1102 produced a larger cataleptic response compared to either 1 mg/kg CP55,940 alone or 1 mg/kg TCS-1102 alone in both sexes (*p* < 0.001) (Figure 1C). Thus, the combination of 1 mg/kg of CP55,940 + 1 mg/kg of TCS-1102 potentiated catalepsy, suggesting additivity between these two drugs. Considering that only co-treatment with the DORA TCS-1102 potentiated catalepsy, these results imply that compared to OX2R antagonism, OX1R antagonism is more critical in potentiating CP55,940-induced catalepsy. In terms of sex differences, males had a larger cataleptic response to the co-treatment with CP55,940 + TCS-1102 compared to females, demonstrating that males are more sensitive to the cataleptic effects of co-administered cannabinoid and DORA (*p* = 0.0032) (Figure 1C).

### Body Temperature

**FIGURE 2:**
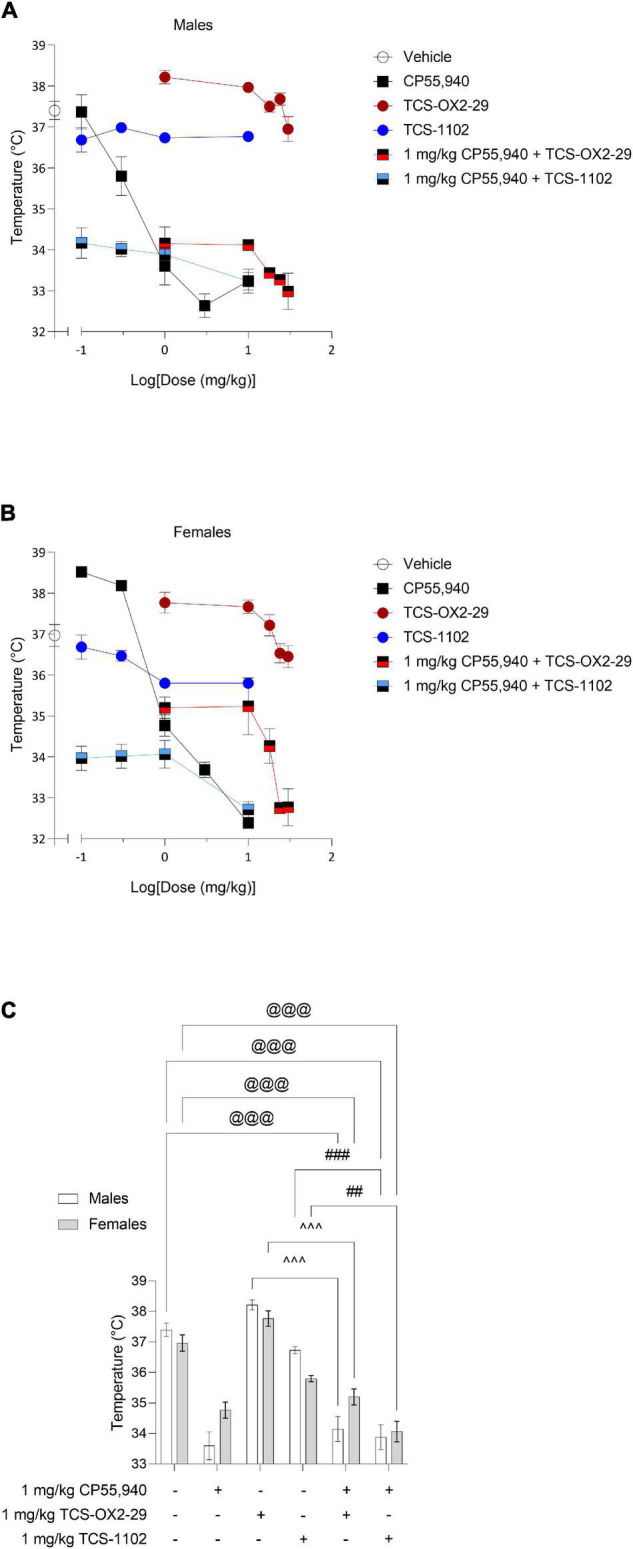
Acute body temperature effects from cannabinoid and orexin drug treatments. Male **(A)** and female **(B)** C57BL/6 mice aged 6–12 weeks were i.p. administered one of the following dose ranges: CP55,940 (0.1–10 mg/kg), TCS-OX2-29 (1–30 mg/kg), TCS-1102 (0.1–10 mg/kg), 1 mg/kg CP55,940 + TCS-OX2-29 (1–30 mg/kg), or 1 mg/kg CP55,940 + TCS-1102 (0.1–10 mg/kg). 15 min post-injections, a rectal thermometer was used to measure internal body temperature. **(C)** Temperature responses were compared within (sex) and between (drugs) the following experimental groups: 1 mg/kg CP55,940, 1 mg/kg TCS-OX2-29, 1 mg/kg TCS-1102, 1 mg/kg CP55,940 + 1 mg/kg TCS-OX2-29, or 1 mg/kg CP55,940 + 1 mg/kg TCS-1102. All catalepsy data are expressed as °C, and as means ± SEM. *n* = 6 for all treatment groups. Significance was calculated using a two-way ANOVA followed by Tukey’s *post hoc* analyses. @@@*p* < 0.001 compared to Vehicle within sexes. ^^^*p* < 0.001 compared between 1 mg/kg TCS-OX2-29 and 1 mg/kg CP55,940 + 1 mg/kg TCS-OX2-29. ##/###*p* < 0.01/0.001 compared between 1 mg/kg TCS-1102 and 1 mg/kg CP55,940 + 1 mg/kg TCS-1102.

With the exception of TCS-1102 treatment in males, all compounds tested produced a dose-dependent decrease in body temperature (Figures 2A,B). Co-treatment with 1 mg/kg CP55,940 + TCS-OX2-29 was a less potent mediator of hypothermia than CP55,940 alone in both sexes (Table 2). Co-treatment with 1 mg/kg CP55,940 + TCS-OX2-29 was more efficacious in producing hypothermia than TCS-OX2-29 alone in both sexes (*p* < 0.001). Co-treatment with 1 mg/kg CP55,940 + TCS-1102 was also more efficacious in producing hypothermia compared to TCS-1102 alone in males (*p* < 0.001) and females (*p* = 0.0049) (Table 2). There were no potency or efficacy differences between any of the other drug treatments, nor between sexes.

**TABLE 2:** *ED*_50_ and *E*_*max*_ values reflecting body temperature responses to cannabinoid and orexin drug treatments.

Treatment	*ED*_50_ (mg/kg) (95% CI)	*E*_*max*_ (°C) ± SEM
	
	Males	
CP55,940	8.8 (4.5–17)	32 ± 0.36
TCS-OX2-29	>30	37 ± 0.36
TCS-1102	n.c.	37 ± 0.090
1 mg/kg CP55,940 + TCS-OX2-29	>30	33 ± 0.45^
1 mg/kg CP55,940 + TCS-1102	12 (5–31)	33 ± 0.87#

	**Females**	
CP55,940	8.6 (4.2–17)	32 ± 0.31
TCS-OX2-29	>30	36 ± 0.27
TCS-1102	10 (4.0–26)	36 ± 0.20
1 mg/kg CP55,940 + TCS-OX2-29	>30	33 ± 0.45^
1 mg/kg CP55,940 + TCS-1102	13 (5.1–32)	33 ± 0.20#

*Data were fit to a three parameter non-linear regression with a system minimum and maximum constrained to 0 and 100, respectively (GraphPad, Prism, v. 8.0).*

*n.c., not converged.*

*For data without a clear dose-response (i.e., “n.c.”), E_max_ is reported as the maximum response observed.*

*Data are expressed as mg/kg with 95% CI or °C ± SEM.*

*^p < 0.05 compared between 1 mg/kg TCS-OX2-29 and 1 mg/kg CP55,940 + 1 mg/kg TCS-OX2-29, and #p < 0.05 compared between 1 mg/kg TCS-1102 and 1 mg/kg CP55,940 + 1 mg/kg TCS-1102, as determined by non-overlapping 95% CI or two-way ANOVA followed by Tukey’s post hoc test.*

*Corresponding graph is presented in Figure 2.*

Co-treatment with 1 mg/kg CP55,940 + 1 mg/kg TCS-1102 produced more hypothermia than 1 mg/kg TCS-1102 alone in both males (*p* < 0.001) and females (*p* = 0.0049) (Figure 2C). Co-treatment with 1 mg/kg CP55,940 + 1 mg/kg TCS-OX2-29 produced a greater decrease in body temperature than 1 mg/kg TCS-OX2-29 alone in both sexes (*p* < 0.001) (Figure 2C). Because these co-treatment effects are not greater than that of CP55,940 alone, hypothermia was likely CP55,940-driven. There were no sex differences in temperature between any of the other 1 mg/kg treatment groups. CP55,940-dependent hypothermia in mice appears to be CBR-dependent and not co-regulated by either OX1R or OX2R, nor sex-dependent.

**FIGURE 3:**
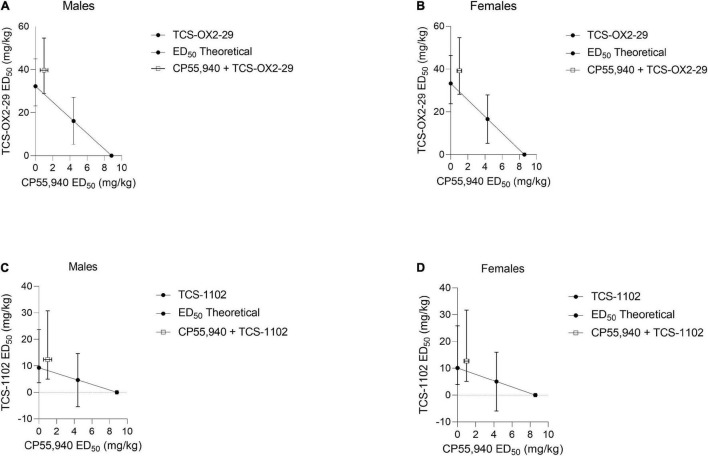
Isobolograms determining drug-drug interactions between cannabinoid and orexin drugs with regards to body temperature. CP55,940 co-treatments with both TCS-OX2-29 **(A,B)** and TCS-1102 **(C,D)** caused non-significant antagonistic body temperature effects in both male **(A,C)** and female **(B,D)** C57BL/6 mice. Data were fit to a three parameter non-linear regression with a system minimum and a system maximum constrained to 0 and 100, respectively. Data are expressed as mg/kg with 95% CI.

Isobolograms comparing compound *ED*_50_ values from Table 2 were constructed for body temperature data (Figure 3). Based on these isobolograms, co-treatments with CP55,940 + TCS-OX2-29 or CP55,940 + TCS-1102 were mapped to the non-significant antagonistic range in both sexes. This is in accordance with the conclusion drawn from Figure 2.

### Anti-nociception

**FIGURE 4:**
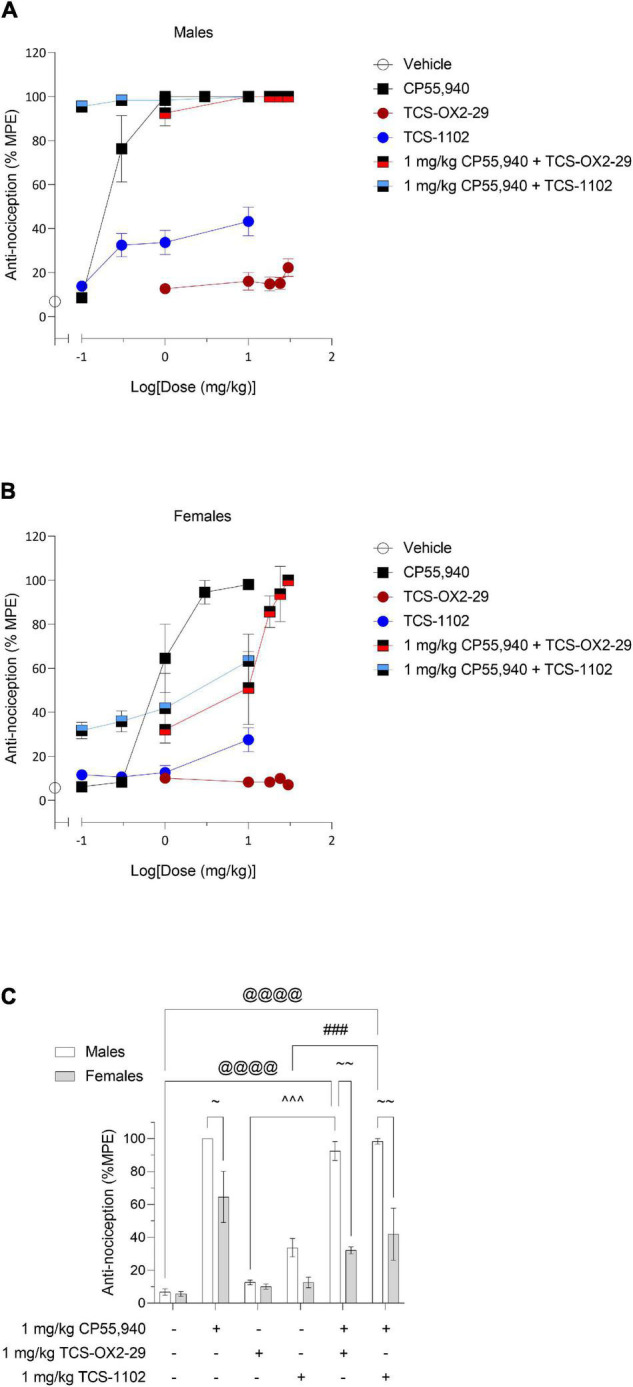
Acute nociceptive effects as a result of cannabinoid and orexin drug treatments. Male **(A)** and female **(B)** C57BL/6 mice aged 6–12 weeks were i.p. administered one of the following dose ranges: CP55,940 (0.1–10 mg/kg), TCS-OX2-29 (1–30 mg/kg), TCS-1102 (0.1–10 mg/kg), 1 mg/kg CP55,940 + TCS-OX2-29 (1–30 mg/kg), or 1 mg/kg CP55,940 + TCS-1102 (0.1–10 mg/kg). 20 min post-injections, all mice underwent the tail flick test to assess anti-thermal nociception. **(C)** Anti–nociceptive responses were compared within (sex) and between (drugs) the following experimental groups: 1 mg/kg CP55,940, 1 mg/kg TCS-OX2-29, 1 mg/kg TCS-1102, 1 mg/kg CP55,940 + 1 mg/kg TCS-OX2-29, or 1 mg/kg CP55,940 + 1 mg/kg TCS-1102. All anti-nociceptive data are expressed as %MPE (MPE = 20 s), and as means ± SEM. *n* = 6 for all treatment groups. Significance was calculated using a two-way ANOVA followed by Tukey’s *post hoc* analyses. @@@*p* < 0.001 compared to Vehicle within sexes. ^^^*p* < 0.001 compared between 1 mg/kg TCS-OX2-29 and 1 mg/kg CP55,940 + 1 mg/kg TCS-OX2-29. ###*p* < 0.001 compared between 1 mg/kg TCS-1102 and 1 mg/kg CP55,940 + 1 mg/kg TCS-1102. ∼∼*p* < 0.01 compared between sexes, within treatment groups.

Dose-response relationships were observed for all groups excluding TCS-OX2-29 in females (Figures 4A,B). For experimental groups without clear dose-response plateaus, *ED*_50_ was estimated to be greater than the maximum dose evaluated (Table 3). Based on this, the co-treatment with 1 mg/kg CP55,940 + TCS-1102 was more potent than 1 mg/kg CP55,940 alone in producing anti-nociception in males (Table 3). The co-treatment with 1 mg/kg CP55,940 + TCS-1102 was more than TCS-1102 alone in females (Table 3). The co-treatment with 1 mg/kg CP55,940 + TCS-OX2-29 was more potent compared to TCS-OX2-29 alone, but less potent compared to CP55,940 alone in females (Table 3). Lastly, the co-treatments with 1 mg/kg CP55,940 + TCS-OX2-29, and 1 mg/kg CP55,940 + TCS/1102, were more efficacious than TCS-OX2-29 alone (*p* < 0.001), and TCS-1102 alone (males: *p* < 0.001, females: *p* = 0.093), respectively (Table 3). No potency or efficacy differences were detected between any other groups, nor between sexes.

**TABLE 3:** *ED*_50_ and *E*_*max*_ values representing anti-nociception responses to cannabinoid and orexin drug treatments.

Treatment	*ED*_50_ (mg/kg) (95% CI)	*E*_*max*_ (%MPE) ± SEM
	
	Males	
CP55,940	0.19 (0.11–0.31)	100
TCS-OX2-29	>30	22 ± 4.0
TCS-1102	7.3 (3.4–15)	41 ± 4.7
1 mg/kg CP55,940 + TCS-OX2-29	n.c.	100^
1 mg/kg CP55,940 + TCS-1102	n.c.	100 ± 1.6#

	**Females**	
CP55,940	0.76 (0.48–1.2)	98 ± 1.9
TCS-OX2-29	n.c.	8.3 ± 1.8
TCS-1102	>10	28 ± 5.5
1 mg/kg CP55,940 + TCS-OX2-29	3.8 (2.0–7.2)*^	100^
1 mg/kg CP55,940 + TCS-1102	1.0 (0.46–2.3)#	72 ± 25#

*Data were fit to a three parameter non-linear regression with a system minimum and maximum constrained to 0 and 100, respectively (GraphPad, Prism, v. 8.0).*

*n.c., not converged.*

*For data without a clear dose-response (i.e., “n.c.”), E_max_ is reported as the maximum response observed.*

*Data are expressed as mg/kg with 95% CI or %MPE ± SEM.*

*p < 0.05 compared to 1 mg/kg CP55,940 within sexes, ^p < 0.05 compared between 1 mg/kg TCS-OX2-29 and 1 mg/kg CP55,940 + 1 mg/kg TCS-OX2-29, and #p < 0.05 compared between 1 mg/kg TCS-1102 and 1 mg/kg CP55,940 + 1 mg/kg TCS-1102, as determined by non-overlapping 95% CI or two-way ANOVA followed by Tukey’s post hoc test.

*Corresponding graph is presented in Figure 4.*

Co-treatment with 1 mg/kg CP55,940 + 1 mg/kg TCS-OX2-29 was associated with a greater anti-nociceptive response than 1 mg/kg TCS-OX2-29 alone in males (*p* < 0.001) (Figure 4C). Moreover, in males, co-treatment with 1 mg/kg CP55,940 + 1 mg/kg TCS-1102 produced a greater anti-nociceptive response than 1 mg/kg TCS-1102 alone (*p* < 0.001) (Figure 4C). There were no differences between compound treatments in females (Figure 4C). Given that neither of the co-treatments in males were more anti-nociceptive than CP55,940 alone, it was concluded that the anti-nociceptive effects of the co-treatments are CP55,940-driven (Figure 4C). With respect to sex differences, CP55,940 alone (*p* = 0.0293) and both co-treatments of CP55,940 + TCS-OX2-29 (*p* < 0.001), and CP55,940 + TCS-1102 (*p* < 0.001), had higher analgesic effects in males compared to females (Figure 4C). Sex differences in nociception were not detected in the orexin receptor antagonist treatments. Therefore, CP55,940-dependent anti-nociception in mice is likely CB1R-dependent and not co-regulated by either OX1R or OX2R, nor sex-dependent.

### Locomotion

**FIGURE 5:**
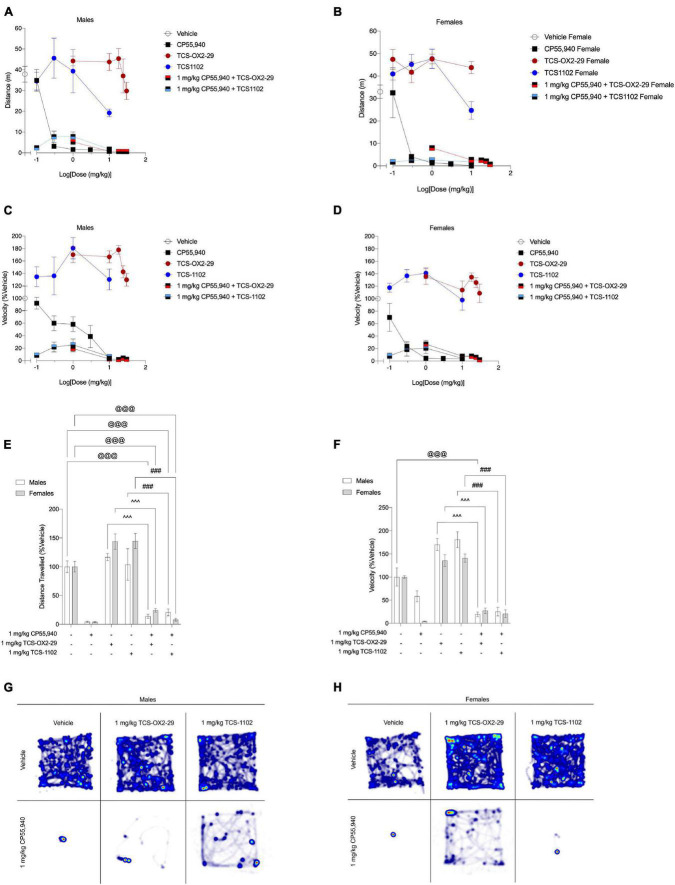
Acute anti-locomotive effects from cannabinoid and orexin drug treatments. **(A–D)** Male and female C57BL/6 mice aged 6–12 weeks were i.p. administered one of the following dose ranges: CP55,940 (0.1–10 mg/kg), TCS-OX2-29 (1–30 mg/kg), TCS-1102 (0.1–10 mg/kg), 1 mg/kg CP55,940 + TCS-OX2-29 (1–30 mg/kg), or 1 mg/kg CP55,940 + TCS-1102 (0.1–10 mg/kg). 25 min post-injections, all mice underwent the open field test to assess locomotion. Distance traveled **(E)** and average velocity **(F)** were compared within (sex) and between (drugs) the following experimental groups: 1 mg/kg CP55,940, 1 mg/kg TCS-OX2-29, 1 mg/kg TCS-1102, 1 mg/kg CP55,940 + 1 mg/kg TCS-OX2-29, or 1 mg/kg CP55,940 + 1 mg/kg TCS-1102. All anti-locomotive data are expressed as m or cm/s, and as means ± SEM. *n* = 6 for all treatment groups. Significance was calculated using a two-way ANOVA followed by Tukey’s *post hoc* analyses. @@@*p* < 0.001 compared to Vehicle within sexes. ^^^*p* < 0.001 compared between 1 mg/kg TCS-OX2-29 and 1 mg/kg CP55,940 + 1 mg/kg TCS-OX2-29. ###*p* < 0.001 compared between 1 mg/kg TCS-1102 and 1 mg/kg CP55,940 + 1 mg/kg TCS-1102. **(G,H)** Representative heat maps illustrating the locomotion of male **(G)** and female **(H)** C57BL/6 mice treated with either 1 mg/kg CP55,940, 1 mg/kg TCS-OX2-29, 1 mg/kg TCS-1102, 1 mg/kg CP55,940 + 1 mg/kg TCS-OX2-29, or 1 mg/kg CP55,940 + 1 mg/kg TCS-1102.

CP55,940, TCS-OX2-29, and TCS-1102 produced dose-dependent decreases in locomotion and velocity in both sexes; however, *ED*_50_ values could not be estimated for 1 mg/kg CP55,940 + TCS-1102 as no plateau was observed (Figure 5 and Table 4). No differences were seen between treatment groups, nor between sexes with regards to the potency in decreasing distance and velocity (Table 4). In both sexes, CP55,940 was more efficacious than TCS-OX2-29 alone and TCS-1102 alone in decreasing distance and velocity (distance in males: *p* = 0.0399; all other groups: *p* < 0.0001) (Table 4). Also in both sexes, co-treatment with 1 mg/kg CP55,940 + TCS-OX2-29 was more efficacious than TCS-OX2-29 alone in decreasing both distance and velocity in both sexes (velocity in females: *p* = 0.0106; all other groups: *p* < 0.0001) (Table 4). In females, co-treatment with 1 mg/kg CP55,940 + TCS-1102 was more efficacious than TCS-110 alone in decreasing distance (*p* = 0.0005) (Table 4). Co-treatment with 1 mg/kg CP55,940 + TCS-1102 was also more efficacious than TCS-1102 alone in decreasing velocity in both sexes (*p* < 0.0001) (Table 4). No other treatment or sex differences were observed in the distance and velocity *E*_*max*_ values.

**TABLE 4:** *ED*_50_ and *E*_*max*_ values summarizing cannabinoid- and orexin-induced locomotion responses.

Treatment	Distance traveled	Average velocity	Distance traveled (%Vehicle)	Average velocity (%Vehicle)
		
	*ED*_50_ (mg/kg) (95% CI)		*E*_max_ ± SEM	
	
	Males			
CP55,940	<0.10	0.51 (0.10–3.0)	3.3 ± 0.41	3.8 ± 1.1
TCS-OX2-29	>30	>30	77 ± 11*	130 ± 10*
TCS-1102	19 (0.10–3.0)	n.c.	51 ± 4.8*	130 ± 17*
1 mg/kg CP55,940 + TCS-OX2-29	n.c.	n.c.	1.0 ± 2.1^	1.7 ± 3.2^
1 mg/kg CP55,940 + TCS-1102	n.c.	n.c.	15 ± 5.3	17 ± 6.6#

	**Females**			
CP55,940	<0.10	<0.10	0	0.68 ± 7.4
TCS-OX2-29	n.c.	n.c.	122 ± 25*	109 ± 15*
TCS-1102	6.1 (0.10–3.0)	n.c.	75 ± 12*	98 ± 16*
1 mg/kg CP55,940 + TCS-OX2-29	n.c.	n.c.	0.63 ± 5.2^	1.2 ± 5.7^
1 mg/kg CP55,940 + TCS-1102	n.c.	n.c.	7.0 ± 1.6#	14 ± 7.0#

*Distance traveled **(a)** and average velocity **(b)** in the open field test were the measures of anti–locomotion.*

*Data were fit to a three parameter non-linear regression with a system minimum and maximum constrained to 0 and 100, respectively (GraphPad, Prism, v. 8.0).*

*For data without a clear dose-response (i.e., “n.c.”), E_max_ is reported as the maximum response observed.*

*Data are expressed as mg/kg with 95% CI or%Vehicle ± SEM.*

*p < 0.05 compared to 1 mg/kg CP55,940 within sexes, ^p < 0.05 compared between 1 mg/kg TCS-OX2-29 and 1 mg/kg CP55,940 + 1 mg/kg TCS-OX2-29, and #p < 0.05 compared between 1 mg/kg TCS-1102 and 1 mg/kg CP55,940 + 1 mg/kg TCS-1102, as determined by non-overlapping 95% CI or two-way ANOVA followed by Tukey’s post hoc test. Corresponding graphs are presented in Figure 5.

Co-treatment with 1 mg/kg CP55,940 + 1 mg/kg TCS-OX2-29 had greater anti-locomotive effects (decreased distance and velocity) compared to TCS-OX2-29 alone in both sexes (*p* < 0.001) (Figures 5E,F). Similarly, co-treatment with 1 mg/kg CP55,940 + 1 mg/kg TCS-1102 had greater anti-locomotive effects (decreased distance and velocity) compared to TCS-1102 alone in both sexes (*p* < 0.001) (Figures 5E,F). Locomotion in the open field test is visualized in representative heat maps (Figures 5G,H). Because no co-treatment exacerbated the anti-locomotive effects of CP55,940 alone, the anti-locomotive effects are likely CP55,940-driven. Lastly, there were no sex differences within any of the drug treatments. Therefore, CP55,940-dependent locomotor effects in mice appear to be CB1R-dependent and not co-regulated by either OX1R or OX2R, nor sex-dependent.

### CB1R and OX1R/OX2R Colocalization

**FIGURE 6:**
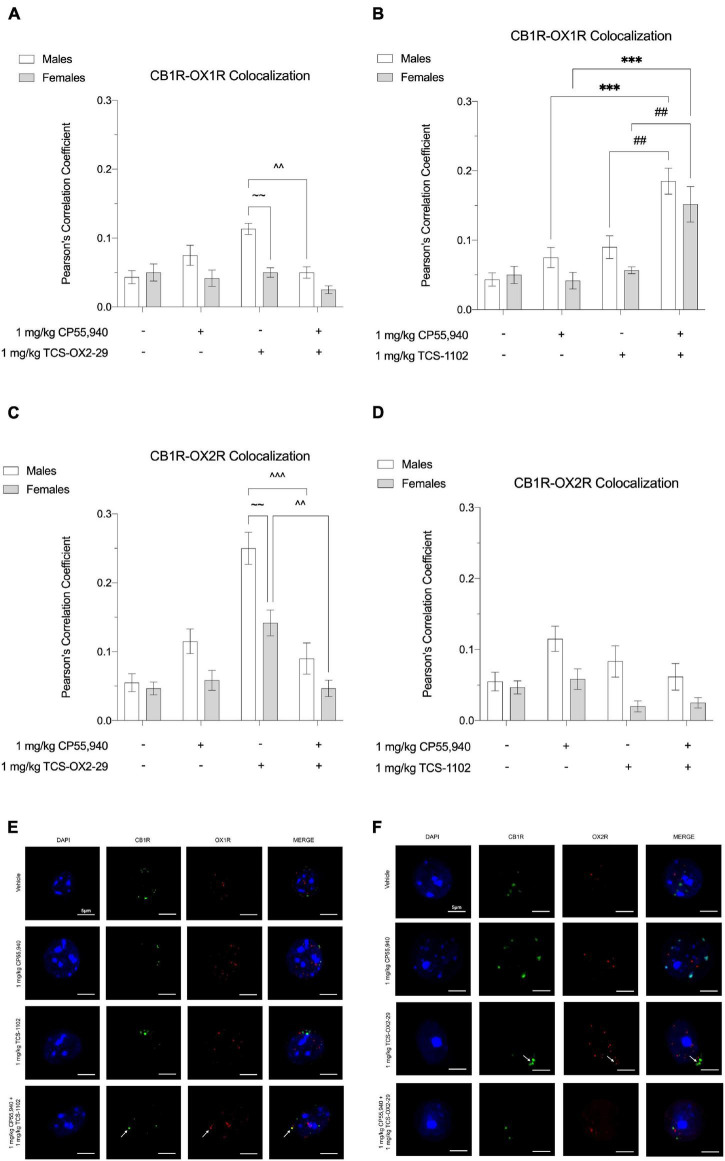
CB1R-OX1R and CB1R-OX2R colocalization in the ventral striatum following cannabinoid and orexin drug treatments. Male and female C57BL/6 mice aged 6–12 weeks were i.p. administered one of the following dose ranges: CP55,940 (0.1–10 mg/kg), TCS-OX2-29 (1–30 mg/kg), TCS-1102 (0.1–10 mg/kg), 1 mg/kg CP55,940 + TCS-OX2-29 (1–30 mg/kg), or 1 mg/kg CP55,940 + TCS-1102 (0.1–10 mg/kg). 30 min post-injections, mice were euthanized, perfused, and their brains collected for immunohistochemistry. Colocalization between CB1R and OX1R **(A,B)** and CB1R and OX2R **(C,D)** was compared within (sex) and between (drugs) the following experimental groups: 1 mg/kg CP55,940, 1 mg/kg TCS-OX2-29, 1 mg/kg TCS-1102, 1 mg/kg CP55,940 + 1 mg/kg TCS-OX2-29, or 1 mg/kg CP55,940 + 1 mg/kg TCS-1102. All colocalization data are expressed as Pearson’s Correlation Coefficients, and as means ± SEM. *n* = 6 (cells) for all treatment groups. Significance was calculated using a two-way ANOVA followed by Tukey’s *post hoc* analyses. ***p < 0.001 compared to 1 mg/kg CP55,940 within sexes. ^^/^^^*p* < 0.01/0.001 compared between 1 mg/kg TCS-OX2-29 and 1 mg/kg CP55,940 + 1 mg/kg TCS-OX2-29. ##*p* < 0.01 compared between 1 mg/kg TCS-1102 and 1 mg/kg CP55,940 + 1 mg/kg TCS-1102. ∼∼*p* < 0.01 compared between sexes, within treatment groups. **(E)** Representative images corresponding to panels **(A,B)** for CB1R-OX1R colocalization. **(F)** Representative images corresponding to panels **(C,D)** for CB1R-OX2R colocalization.

**FIGURE 7:**
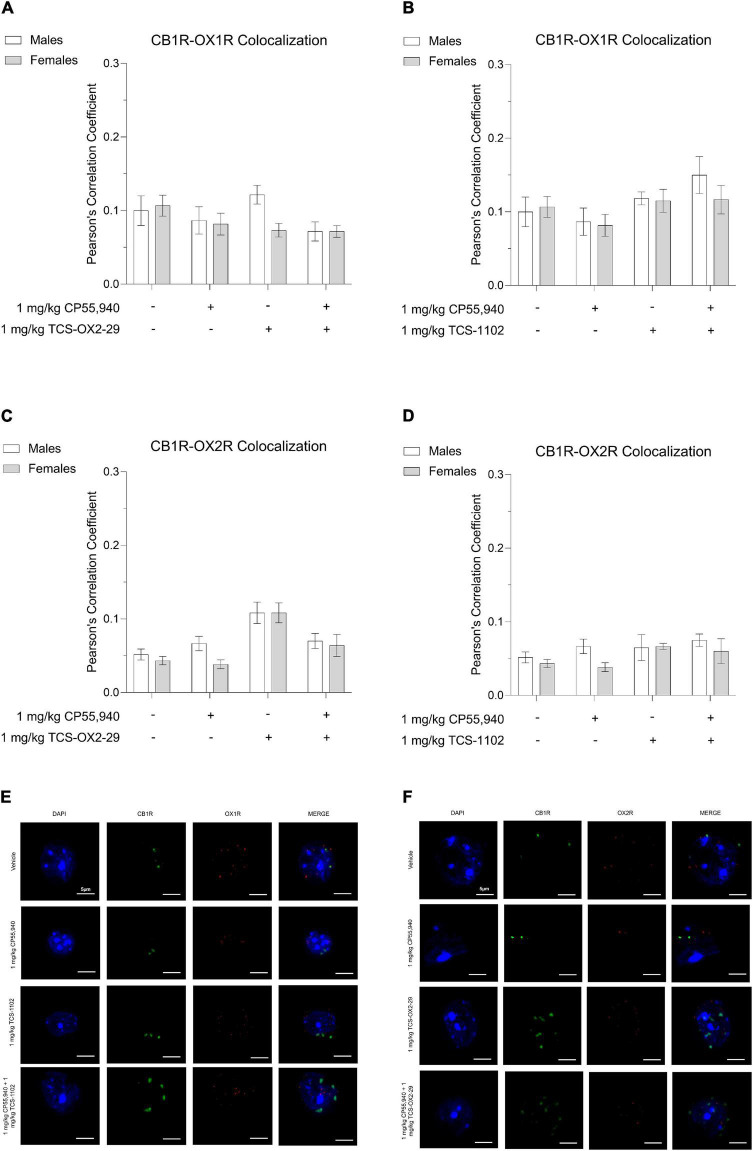
CB1R-OX1R and CB1R-OX2R colocalization in the primary motor cortex following cannabinoid and orexin drug treatments. Male and female C57BL/6 mice aged 6–12 weeks were i.p. administered one of the following dose ranges: CP55,940 (0.1–10 mg/kg), TCS-OX2-29 (1–30 mg/kg), TCS-1102 (0.1–10 mg/kg), 1 mg/kg CP55,940 + TCS-OX2-29 (1–30 mg/kg), or 1 mg/kg CP55,940 + TCS-1102 (0.1–10 mg/kg). 30 min post-injections, mice were euthanized, perfused, and their brains collected for immunohistochemistry. Colocalization between CB1R and OX1R **(A,B)** and CB1R and OX2R **(C,D)** was compared within (sex) and between (drugs) the following experimental groups: 1 mg/kg CP55,940, 1 mg/kg TCS-OX2-29, 1 mg/kg TCS-1102, 1 mg/kg CP55,940 + 1 mg/kg TCS-OX2-29, or 1 mg/kg CP55,940 + 1 mg/kg TCS-1102. All colocalization data are expressed as Pearson’s Correlation Coefficients, and as means ± SEM. *n* = 6 (cells) for all treatment groups. Significance was calculated using a two-way ANOVA followed by Tukey’s *post hoc* analyses. **(E)** Representative images corresponding to panels **(A,B)** for CB1R-OX1R colocalization. **(F)** Representative images corresponding to panels **(C,D)** for CB1R-OX2R colocalization.

Thirty min post-injection, brain tissue was collected from the following drug treatment groups: 1 mg/kg CP55,940, 1 mg/kg TCS-OX2-29, 1 mg/kg TCS-1102, 1 mg/kg CP55,940 + 1 mg/kg TCS-OX2-29, and 1 mg/kg CP55,940 + 1 mg/kg TCS-1102. Immunohistochemical experiments focused on two brain regions: (1) ventral striatum, a brain region thought to facilitate cataleptic effects (Turski et al., 1984; Ossowska et al., 1990; Anderson et al., 1996) and (2) primary motor cortex, a region that largely initiates and modulates locomotion (Sahni et al., 2020). In the ventral striatum, more whole-cell CB1R-OX1R colocalization was observed in males treated with 1 mg/kg TCS-OX2-29 compared to males treated with the combination of 1 mg/kg CP55,940 + 1 mg/kg TCS-OX2-29; this difference was not seen in treatment-matched females (Figure 6A). Between males and females treated with 1 mg/kg TCS-OX2-29, ventral striatum tissue from males had higher CB1R-OX1R colocalization (Figure 6A). In both sexes, CB1R-OX1R colocalization in the ventral striatum was greater following 1 mg/kg CP55,940 + 1 mg/kg TCS-1102 compared to both 1 mg/kg CP55,940 alone and 1 mg/kg TCS-1102 alone (Figure 6B); this is further illustrated by representative images (Figure 6E). No sex differences were observed within the TCS-1102-treated groups with regards to CB1R-OX1R colocalization in the ventral striatum (Figure 6B). In the primary motor cortex, there were no significant differences in CB1R-OX1R colocalization between any of the experimental groups (Figures 7A,B,E).

In both sexes, CB1R-OX2R colocalization in the ventral striatum was higher in 1 mg/kg TCS-OX2-29-treated mice compared to mice co-treated with 1 mg/kg CP55,940 + 1 mg/kg TCS-OX2-29 (Figure 6C). Compared to females, ventral striatum tissue from males treated with 1 mg/kg TCS-OX2-29 had larger CB1R-OX2R colocalization (Figure 6C); this is demonstrated in the representative images (Figure 6F). There were no differences in ventral striatum CB1R-OX2R colocalization within or between any of the groups treated with TCS-1102 (Figure 6D). Moreover, the primary motor cortex did not display differences in CB1R-OX2R colocalization between any of the experimental groups (Figures 7C,D,F). To summarize, 1 mg/kg CP55,940 + 1 mg/kg TCS-1102 was the only combination treatment that displayed higher CB1R-OX1R colocalization in the ventral striatum compared to its constituent drugs alone (Figure 6B). This supports the tetrad data, in which this combination treatment produced more catalepsy than each of its constituent drugs (Figure 1C). As for CB1R-OX2R colocalization in the ventral striatum, 1 mg/kg TCS-OX2-29 co-treatment was associated with more colocalization than the co-treatment with CP55,940 and TCS-OX2-29 (Figure 6C). Compared to OX2R antagonism, OX1R antagonism and subsequent changes in CB1R-OX1R colocalization following co-treatment with the CB1R agonist CP55,940 and the DORA TCS-1102 are likely to be the potentiators of catalepsy. None of the drug treatments produced significant CB1R-OX1R nor CB1R-OX2R colocalization changes in the primary motor cortex, which supports the lack of the CP55,940 and TCS-1102 additivity with regards to reduced movement in the open field test.

## Discussion

To date, the majority of endocannabinoid and orexin interaction studies have either characterized their molecular or physical interactions *in vitro*, or investigated their physiological interdependence in complex disease models related to sleep, appetite, and reward. One other study has assessed their dual modulation of body temperature, pain, locomotion, anxiety, and memory in healthy and transgenic male mice (Flores et al., 2016). The current study aimed to not only measure catalepsy alongside other measures of the tetrad, but utilize a full CB1R agonist and a clinically relevant DORA in both male and female mice. Compared to the CB1R partial agonist Δ^9^-THC, which was used by Flores et al. (2016), CP55,940 is a full agonist of CB1R (Howlett and Abood, 2017) that is well-documented to produce more potent and efficacious responses *in vitro* and *in vivo* (Patel et al., 2020; Zagzoog et al., 2020; Henderson-Redmond et al., 2021). In the current study, CP55,940 (0.1–10 mg/kg) produced similar dose-dependent sedative effects in both males and females.

The OX2R antagonist TCS-OX2-29 and the DORA TCS-1102 were the orexin receptor compounds used in the current study. Treatment with either orexin receptor antagonist alone was associated with hypothermic, anti-nociceptive, and anti-locomotive effects of smaller magnitude than that of CP55,940. TCS-1102 was generally more efficacious compared to TCS-OX2-29, suggesting either a greater role for OX1R or both orexin receptor subtypes in controlling body temperature, nociception, and locomotion. In a previous study, OX1R antagonism *via* SB-334867 was found to potentiate Δ^9^-THC-induced hypothermia, anti-nociception, and anxiolytic-like effects, while OX2R antagonism by TCS-OX2-29 was not (Flores et al., 2016). The current study was more focused on evaluating a clinically relevant DORA in conjunction with a cannabinoid. DORAs such as Suvorexant and Lemborexant are used for insomnia as they cause sedation by blocking the arousing effects of endogenous orexins (Yoshimichi et al., 2001; Patel et al., 2015; Herring et al., 2019). Comparisons between the orexin receptor subtypes in the context of the sleep-wake cycle have determined that although OX2R is more critical in inducing arousal, OX1R plays additional roles in promoting and maintaining wakefulness (Mieda et al., 2011; Mang et al., 2012).

Aside from catalepsy, co-manipulation of both cannabinoid and orexin receptors did not alter responses in the tetrad battery of assays compared to cannabinoid agonism alone. Thus, CP55,940-induced hypothermia, anti-nociception, and anti-locomotion are not regulated by OX1R- nor OX2R. Based on the dose-response curves for the co-treatments of CP55,940 and each of the orexin receptor antagonists, it can be confirmed that additivity between these two drug types with regards to hypothermia, anti-nociception, and anti-locomotion, does not exist at any dose. In terms of non-heterodimerized, non-interacting cannabinoid and orexin receptors, these results suggest that CBRs have a greater physiological role than orexin receptors in (1) the preoptic anterior hypothalamus and control of body temperature (Rawls et al., 2002), (2) spine and periaqueductal gray area underlying nociception (Freund et al., 2003; Walker and Hohmann, 2005), and (3) cortical regions in modulating locomotion (Polissidis et al., 2013). CB1R remains one of the most abundant GPCRs in the CNS (Mackie, 2005; Kano et al., 2009). Orexin receptor mRNA is also found in these regions (Trivedi et al., 1998; Marcus et al., 2001), however, there are no studies comparing the relative abundance of cannabinoid and orexin receptors in the same samples.

Unlike body temperature, nociception, and locomotion, co-treatment with CP55,940 and TCS-1102 produced longer-lasting catalepsy than CP55,940 or TCS-1102 alone, demonstrating additivity where each drug had an equal role in producing catalepsy. This was not observed for the combination of CP55,940 and TCS-OX2-29, suggesting that OX1R antagonism is more critical in potentiating CP55,940-induced catalepsy. Additivity between these two drugs may be better explained by regional differences in CB1R-OX1R and CB1R-OX2R interactions or heterodimerization in the ventral striatum, a sub-cortical region that expresses all three receptor subtypes to modulate catalepsy (Turski et al., 1984; Ossowska et al., 1990; Flores et al., 2013). Beyond the ventral striatum, orexin receptors are sparse in the dorsal striatum and more densely expressed in the ventral striatum (Hervieu et al., 2001; Marcus et al., 2001; Mackie, 2005; Flores et al., 2013). Our current study used colocalization as a proof-of-concept for the main behavioral data; thus aimed to efficiently gather data from brain regions that are well-documented co-expressed both receptor types. Moreover, the nucleus accumbens and the olfactory tubercle (which composes the ventral striatum) expresses both CB1R and OX2R (Flores et al., 2013). Although the nucleus accumbens is known to process emotions and reward, it also integrates emotional or motivational stimuli as it relates to sedation (Valencia Garcia and Fort, 2018). Anti-locomotion is a focal point in our current study because both cannabinoids and orexin drugs cause sedation. The olfactory tubercle processes incoming sensory information which may include rewarding stimuli (Wesson and Wilson, 2011; Murata, 2020). When CBRs and OX1R were co-manipulated in the current study, there was more CB1R-OX1R colocalization in the ventral striatum. Similar observations have been made in recombinant cell cultures, where the OX1R antagonist SB-674042 and CB1R antagonist SR141716A alone caused relocalization of OX1R and CB1R together (Ellis et al., 2006). Neither of these antagonists had significant affinities for the other receptor type, suggesting that inhibiting one receptor type caused relocalization of the other by physical proxy (Ellis et al., 2006).

Sex differences were observed in two scenarios. First, male C57BL/6 mice were more sensitive to the cataleptic effects of 1 mg/kg C55,940 + 1 mg/kg TCS-1102 compared to treatment-matched females. Sex differences in catalepsy were not detected in the TCS-OX2-29-administered groups, indicating that males are more sensitive than females to simultaneous CBR and OX1R- but not OX2R-manipulation. Closer examination of the receptor colocalization results revealed that in the absence of cannabinoid and orexin drug administration, males and females had similar levels of CB1R-OX1R and CB1R-OX2R expression in the ventral striatum. Following treatment with TCS-OX2-29 alone, cells from the ventral striatum of males had higher levels of CB1R-OX1R/OX2R colocalization compared to females. In all other drug treatments, no significant difference in cannabinoid and orexin receptor colocalization was observed between sexes. Male rodents have lower hypothalamic mRNA levels of OXA and OXB precursor, prepro-orexin (Jöhren et al., 2002), as well as less basal activation of OXA containing lateral hypothalamic neurons (Grafe et al., 2017). Although female rodents are reported to have higher orexigenic functioning (Grafe and Bhatnagar, 2020), males may have greater expression and function of orexin receptors that interact, or are heterodimerized with CB1R. This may result in males being more sensitive to dual cannabinoid and orexin drug effects compared to females.

The second sex difference observed was within the tail flick test. Males were more sensitive to the analgesic effects of CP55,940 alone, as well as both combination drug treatments. Most behavioral studies have reported that females are more sensitive to the cataleptic and anti-nociceptive effects of phyto- and synthetic cannabinoid agonists (Tseng and Craft, 2001; Wiley et al., 2017). With regards to brain region-specific cannabinoid receptor expression and function, CB1R density is greater in the prefrontal cortex of male versus female rats (Castelli et al., 2014). Males also display higher CB1R binding in limbic regions such as the striatum (Rodríguez de Fonseca et al., 1994). It remains unclear how these molecular data translate to sex-dependent behavioral outcomes, as these types of experiments have never been conducted nor correlated in the same sample or study. Preclinical cannabinoid research is seeing more inclusion of female animals; however, endocannabinoid-sex hormone interactions are more complex than simply comparing testosterone-dominant males and estrogen-dominant females (Struik et al., 2018). The latter undergo hypothalamic-pituitary-gonadal-driven ovulation cycles that cause significant fluctuations in circulating estrogens and progestins. The mouse estrus cycle spans 4 days, throughout which these hormone levels influence endocannabinoid activity and physiological response to cannabinoids (Rodríguez de Fonseca et al., 1994; Wakley and Craft, 2011). For example, female rats in estrus are significantly less sensitive to the sedative and analgesic effects of systemic Δ^9^-THC compared to female rats in late proestrus (Wakley and Craft, 2011). Estrus cycle-dependent differences in cannabinoid and orexin drug responses are being investigated in a forthcoming proof-of-concept study.

Cannabis is one of the most highly consumed psychoactive drugs in the world (World Health Organization [WHO], 2016). Many people self-medicate with cannabis to induce relaxation; a subset of these individuals may co-administer cannabis with prescribed DORAs for insomnia. Sleep is a complicated behavior based on multiple physiological factors. Although both cannabinoid receptor agonists and orexin receptor antagonists individually promote sleep, they may differentially affect the conditions for sleep when combined. The current study found that catalepsy was the only tetrad measure equally potentiated by both drugs. Moreover, OX1R antagonism, rather than OX2R antagonism, resulted in increased CB1R-OX1R colocalization in the ventral striatum underlying this cataleptic additivity. The growing use of cannabinoids warrants more research in the area of cannabinoid-drug interactions. Knowledge of cannabinoid receptor heterodimerization with other GPCRs is key in understanding these pharmacodynamic interactions.

## Data Availability Statement

The raw data supporting the conclusions of this article will be made available by the authors, without undue reservation.

## Ethics Statement

The animal study was reviewed and approved by Animal Research Ethics Board and the Scientific Merit Review Committee for Animal Behaviour at the University of Saskatchewan.

## Author Contributions

HJK designed and executed the experiments, analyzed the data, as well as wrote and edited the manuscript. AZ, AMS, UCE, MJB, and TH assisted with *in vivo* experiments and edited the manuscript. RBL aided in the design of the experiments, analyzed the data, and edited the manuscript.

## Conflict of Interest

The authors declare that the research was conducted in the absence of any commercial or financial relationships that could be construed as a potential conflict of interest.

## Publisher’s Note

All claims expressed in this article are solely those of the authors and do not necessarily represent those of their affiliated organizations, or those of the publisher, the editors and the reviewers. Any product that may be evaluated in this article, or claim that may be made by its manufacturer, is not guaranteed or endorsed by the publisher.
